# Therapeutic strategies against biofilm-associated multidrug-resistant *Klebsiella pneumoniae* and *Acinetobacter baumannii* in ventilator-associated pneumonia

**DOI:** 10.3389/fmicb.2026.1904655

**Published:** 2026-08-05

**Authors:** Pooja Rao, Jamuna Bai Aswathanarayan, SubbaRao V. Madhunapantula, Ravishankar Rai Vittal

**Affiliations:** 1 Department of Microbiology, School of Life Sciences, JSS Academy of Higher Education and Research, Mysuru, India; 2 Department of Biochemistry, JSS Medical College, Mysuru, India; 3 Department of Studies in Microbiology, University of Mysuru, Mysuru, India

**Keywords:** *Acinetobacter baumannii*, anti-biofilm therapeutics, biofilm-mediated persistence, *Klebsiella pneumoniae*, ventilator-associated pneumonia

## Abstract

Ventilator-associated pneumonia remains one of the most common and severe healthcare associated infections in critically ill patients receiving mechanical ventilation. Among the predominant causative pathogens, multidrug-resistant *Klebsiella pneumoniae* and *Acinetobacter baumannii* pose a major clinical challenge due to their extensive antimicrobial resistance, environmental persistence, and ability to form biofilms on endotracheal tubes and other medical devices. Biofilm formation is central to VAP pathogenesis, facilitating bacterial adhesion, immune evasion, reduced antimicrobial penetration, and protective niches that support metabolic dormancy, persister-cell formation, and horizontal gene transfer. These mechanisms promote bacterial survival, recurrent infection, prolonged hospitalization, increased healthcare costs, and mortality. Resistance in *K. pneumoniae* and *A. baumannii* is mediated by multiple mechanisms, including β-lactamase production, efflux pump overexpression, reduced outer membrane permeability, target-site modification, and acquisition of mobile resistance determinants. The interaction between AMR and biofilm- associated persistence substantially compromises antimicrobial efficacy, particularly against carbapenem-resistant strains. Although recent advances have expanded treatment options, outcomes remain suboptimal in established biofilm-associated infections. This review examines the epidemiology, clinical burden, resistance mechanisms, biofilm mediated persistence, and host-pathogen interactions underlying MDR *K. pneumoniae* and *A. baumannii* associated VAP. Particular emphasis is placed on current therapeutic approaches and emerging anti-biofilm strategies, including antimicrobial-coated devices, nanotechnology-based interventions, matrix-disrupting enzymes, antimicrobial peptides, and anti-virulence approaches targeting quorum sensing and biofilm maturation. Understanding the interplay between AMR, biofilm persistence, and therapeutic failure is critical for developing more effective strategies to prevent and manage MDR-VAP.

## 1 Introduction

Antimicrobial resistance (AMR) has emerged as one of the most significant global public health threats of the 21st century, compromising the effectiveness of antibiotics and increasingly limiting treatment options for severe bacterial infections (Iskandar et al., 2021). The rapid emergence of multidrug-resistant (MDR), extensively drug-resistant (XDR), and pan-drug-resistant (PDR) pathogens has transformed many previously manageable infections into major clinical challenges associated with prolonged hospitalization, increased healthcare costs, and elevated mortality (Murray et al., 2022; Rupp et al., 2024). Among the ESKAPE pathogens (*Enterococcus faecium, Staphylococcus aureus, Klebsiella pneumoniae, Acinetobacter baumannii, Pseudomonas aeruginosa*, and *Enterobacter* spp.), *K pneumoniae* and *A. baumannii* are of particular concern because of their remarkable ability to acquire antimicrobial resistance, persist in healthcare environments, and cause severe device-associated infections, particularly ventilator-associated pneumonia (VAP) (Kharat et al., 2024; Hamidian and Nigro, 2019; Miller and Arias, 2024). VAP remains one of the most common healthcare-associated infections among mechanically ventilated patients in intensive care units (ICUs). The incidence varies considerably across healthcare systems, ranging from approximately 6% to 52%, depending on diagnostic criteria, duration of ventilation, patient populations, and regional healthcare practices (Kharel et al., 2021; Rana et al., 2025). In India, reported VAP incidence ranges from 30% to 45%, with *K. pneumoniae* and *A. baumannii* consistently identified among the predominant etiological agents (Kar et al., 2024; Gupta et al., 2025). Globally, outbreaks involving MDR Gram-negative pathogens, particularly during the COVID-19 pandemic, further highlighted the vulnerability of mechanically ventilated patients to device-associated infections (Loyola-Cruz et al., 2023; Meshram et al., 2025).

The clinical challenge posed by these pathogens extends beyond antimicrobial resistance alone. Carbapenem-resistant *K. pneumoniae* (CRKP) and Carbapenem-resistant *A. baumannii* (CRAB) are recognized by the World Health Organization (WHO) as critical priority pathogens because of their limited therapeutic options and high mortality (WHO, 2024). Resistance is mediated through multiple mechanisms, including production of extended-spectrum β-lactamases (ESBLs) and carbapenemases such as *K. pneumoniae* carbapenemase (KPC), New Delhi metallo-β-lactamase (NDM), Verona integron-encoded metallo-β-lactamase (VIM), imipenemase (IMP), and oxacillinase (OXA)-type enzymes, together with reduced membrane permeability, efflux pump overexpression, and target-site modification (Bush and Bradford, 2020; Lawrence et al., 2024; Puri et al., 2025). These resistance determinants substantially limit the effectiveness of last-resort antimicrobial agents and contribute to poor clinical outcomes (Hamidian and Nigro, 2019; Yang et al., 2023).

A defining feature of *K. pneumoniae* and *A. baumannii* is their ability to establish biofilms on endotracheal tubes (ETTs) and other medical devices (Marcut et al., 2023; Mishra et al., 2024). Within these highly organized microbial communities, bacteria are embedded in a self-produced extracellular polymeric substance (EPS) matrix composed of polysaccharides, proteins, lipids, and extracellular DNA (eDNA) (Deshmukh-Reeves et al., 2025; Flemming et al., 2025). Biofilm-associated growth restricts antimicrobial penetration, promotes metabolic dormancy and persister-cell formation, facilitates horizontal gene transfer (HGT), and impairs host immune clearance, collectively contributing to persistent infection and therapeutic failure, (Kunnath et al., 2024; Sharif and Yadav, 2025).

Despite advances in antimicrobial therapy and infection prevention, effective management of biofilm-associated MDR-VAP remains challenging because antimicrobial resistance, biofilm-mediated persistence, and host-pathogen interactions function as interconnected drivers of therapeutic failure rather than independent processes (Chen et al., 2022; Mitache et al., 2025). Therefore, this review provides a comprehensive overview of the clinical burden of MDR *K. pneumoniae* and *A. baumannii* in VAP, the antimicrobial resistance mechanisms and biofilm-associated processes underlying persistent infection, host-pathogen interactions that contribute to disease progression, and current international guideline-based management strategies (Chandrashekar et al., 2026). It further examines emerging anti-biofilm therapeutic approaches, including antimicrobial-coated devices, nanotechnology-based interventions, bacteriophage therapy and bacteriophage-derived depolymerases, matrix-disrupting enzymes, antimicrobial peptides, anti-virulence strategies, and combination therapies (Upmanyu et al., 2024; Berry et al., 2024; Zavialov et al., 2025). A central perspective of this review is that antimicrobial resistance, biofilm-mediated persistence, and host-pathogen interactions should be viewed as interconnected drivers of therapeutic failure rather than independent processes. By integrating these interrelated mechanisms, this review provides a conceptual framework for understanding the limitations of conventional therapies and the rationale for the development of emerging anti-biofilm strategies for the management of MDR-VAP caused by *K. pneumoniae* and *A. baumannii* (Chen et al., 2022).

## 2 Literature search strategy

This review summarizes the current evidence on biofilm-associated MDR *K. pneumoniae* and *A. baumannii* in VAP. A comprehensive literature search was performed using PubMed-MEDLINE, Scopus, Web of Science, and Google Scholar to identify relevant studies published primarily between 2015 and 2025. Key earlier studies were included to provide historical context and explainthe basic concepts of antimicrobial resistance, biofilm biology and VAP pathogenesis. Search terms included combinations of “ventilator-associated pneumonia,” “*Klebsiella pneumoniae*,” “*Acinetobacter baumannii*,” “biofilm,” “antimicrobial resistance,” “carbapenem resistance,” “endotracheal tube,” “anti-biofilm therapy,” “nanoparticles,” “antimicrobial peptides,” “phage therapy,” “quorum sensing,” and “medical device coatings.” Reference list of selected publications were also searched to identify additional relevant articles.

Original research articles, systematic reviews, meta-analyses, clinical guidelines, and authoritative reports from organizations such as the World Health Organization (WHO), Centers for Disease Control and Prevention (CDC), Infectious Diseases Society of America (IDSA), American Thoracic Society (ATS), and European Society of Clinical Microbiology and Infectious Diseases (ESCMID) were considered. Priority was given to peer-reviewed studies that addressed clinically relevant aspects of MDR pathogens, biofilm formation, host-pathogen interactions, and emerging therapeutic strategies. Publications lacking sufficient methodological detail or relevance to the review objectives were not included.

## 3 Clinical burden of MDR *Klebsiella pneumoniae* and *Acinetobacter baumannii* in ventilator-associated pneumonia

### 3.1 Epidemiology and prevalence

VAP remains one of the most frequent and severe healthcare-associated infections in mechanically ventilated patients, particularly within ICUs. The incidence of VAP varies substantially across healthcare systems and patient populations, ranging from approximately 6% to 52% depending on diagnostic criteria, duration of ventilation, ICU infrastructure, and regional antimicrobial stewardship practices (Kharel et al., 2021; Rana et al., 2025). The burden is especially prominent in low and middle-income countries, where high antibiotic consumption, limited infection control resources, and overcrowded ICUs contribute to increased transmission of MDR pathogens (Chandrashekar et al., 2026). Among the ESKAPE pathogens, *K. pneumoniae* and *A. baumannii* have emerged as dominant etiological agents of VAP because of their remarkable environmental persistence, rapid acquisition of antimicrobial resistance determinants, and strong ability to colonize ETTs and other medical devices through biofilm formation (Hamidian and Nigro, 2019; Marcut et al., 2023). In several ICU surveillance studies, Gram-negative non-fermenting bacilli and Enterobacterales account for the majority of VAP-associated isolates, with *K. pneumoniae* and *A. baumannii* consistently among the most prevalent pathogens (Meshram et al., 2025; Kumar et al., 2025). In India, the prevalence of MDR *K. pneumoniae* and *A. baumannii* in ventilated patients is particularly concerning. Studies investigating VAP in Indian ICUs report incidence rates ranging from 30% to 45%, with *K. pneumoniae* and *A. baumannii* frequently identified as the predominant causative agents (Kar et al., 2024; Gupta et al., 2025). In Thanjavur, Tamil Nadu, early-onset VAP caused by *K. pneumoniae* was reported in up to 38.9% of ICU patients (Kumar et al., 2025). Similarly, a surveillance study conducted in Bhubaneswar, Odisha identified *K. pneumoniae* in 55.9% of VAP-associated isolates and *A. baumannii* in 17% of isolates, with many strains exhibiting MDR patterns (Rana et al., 2025). Surveillance data from tertiary-care hospitals across India further indicate increasing carbapenem resistance among Gram-negative ESKAPE pathogens, reflecting widespread dissemination of resistance determinants under strong antibiotic selection pressure (Mogasale et al., 2021; Chandrashekar et al., 2026).

Globally, CRKP and CRAB are now recognized as major ICU-associated threats. MDR rates of *A*. *baumannii* frequently exceed 60%−80% in parts of Asia, the Middle East, and Southern Europe, where CRAB has become endemic in many healthcare systems (Manjusha et al., 2023; Scribano et al., 2024). Likewise, the prevalence of CRKP has risen substantially over the past decade, with carbapenem resistance approaching 30% globally and exceeding 60% in some high-burden regions (Asokan et al., 2025; Zanella et al., 2026; CDC, 2026). The spread of resistance is largely mediated by mobile genetic elements carrying carbapenemase genes such as *blaKPC, blaNDM*, and *blaOXA*-48 in *K. pneumoniae*, and *OXA*-type carbapenemases including *OXA*-23, *OXA*-24/40, and *OXA*-58 in *A. baumannii* (Hamidian and Nigro, 2019; Bush and Bradford, 2020). The COVID-19 pandemic further intensified the burden of MDR-VAP pathogens. Several healthcare systems reported increased outbreaks of *A. baumannii* and *P. aeruginosa* among mechanically ventilated COVID-19 patients, largely driven by prolonged ventilation, broad-spectrum antibiotic exposure, overwhelmed healthcare infrastructure, and increased device utilization (Loyola-Cruz et al., 2023). These observations reinforced the role of MDR Gram-negative pathogens as persistent opportunistic threats in critically ill patients and highlighted the importance of strengthened infection control and antimicrobial stewardship programs in ICUs.

**Figure 1:**
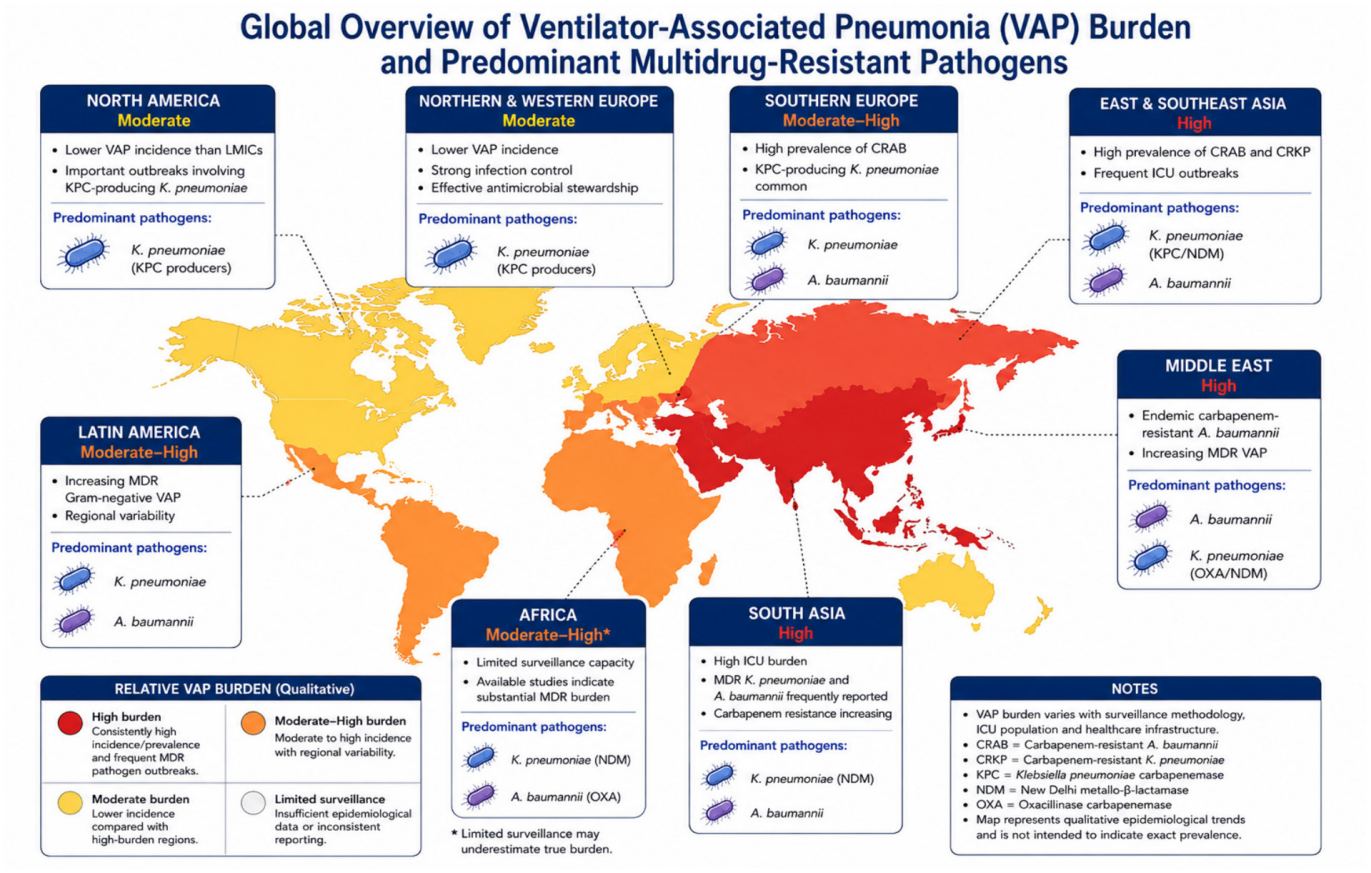
Global epidemiological overview of ventilator-associated pneumonia burden, highlighting regional variations in the prevalence of MDR *K. pneumoniae* and *A. baumannii*.

Although *K. pneumoniae* and *A. baumannii* account for a substantial proportion of MDR-VAP cases, other Gram-negative pathogens, particularly *P*. *aeruginosa* and *Enterobacter* spp., also contribute significantly to the global burden of VAP (Rana et al., 2025). *P*. *aeruginosa* remains one of the most frequently isolated VAP pathogens worldwide and is associated with high mortality, prolonged mechanical ventilation, and intrinsic resistance to multiple antimicrobial agents (Kharat et al., 2024). Similarly, *Enterobacter* spp., while less frequently encountered, have emerged as important opportunistic pathogens in ICU settings contributing to the increasing prevalence of ESBL and Ampicillinase C (AmpC) producing strains, which further complicate therapeutic management (Miller and Arias, 2024). The prevalence of these pathogens varies considerably across geographic regions, patient populations and local antimicrobial resistance patterns, underscoring the dynamic epidemiology of VAP (WHO, 2024; Zirpe et al., 2025; CDC, 2026) as shown in Figure 1.

### 3.2 Therapeutic challenges

The clinical impact of MDR *K. pneumoniae* and *A. baumannii* infections extends far beyond increased infection rates. VAP caused by these pathogens is strongly associated with prolonged mechanical ventilation, extended ICU and hospital stays, increased healthcare expenditure, and significantly higher mortality compared with infections caused by susceptible pathogens (Kharel et al., 2021; Miller and Arias, 2024). In severe infections caused by MDR *A. baumannii*, mortality rates may reach 50%−70%, particularly among critically ill or immunocompromised patients (Miller and Arias, 2024). Similarly, carbapenem-resistant *K. pneumoniae* infections are associated with poor clinical outcomes because of delayed effective therapy and limited treatment options (Zirpe et al., 2025). Therapeutic management of these infections has become increasingly difficult because resistance mechanisms frequently coexist within the same strain, resulting in MDR and XDR phenotypes (Yang et al., 2023). Carbapenemases such as KPC, NDM, and OXA-type enzymes substantially reduce the efficacy of last-line β-lactam antibiotics, while additional mechanisms including efflux pumps, porin loss, and target-site modification further restrict antimicrobial susceptibility (Hamidian and Nigro, 2019; Bush and Bradford, 2020). Consequently, treatment often relies on older or more toxic agents such as polymyxins, tigecycline, aminoglycosides, or combination regimens with variable clinical success (Mantzana et al., 2023; Mackow and van Duin, 2024). The challenge is further enhanced by biofilm formation on ETTs and other medical devices. Biofilm-associated bacteria exhibit markedly increased tolerance to antibiotics and host immune responses because the EPS matrix restricts antimicrobial penetration, promotes metabolic dormancy, and facilitates horizontal transfer of resistance genes (Bollen et al., 2023; Sharif and Yadav, 2025). As a result, infections frequently persist despite aggressive antimicrobial therapy and may recur even after microbiological clearance (Marcut et al., 2023). In many cases, successful management requires removal or replacement of colonized devices in addition to prolonged antimicrobial treatment (Kunnath et al., 2024). Recognition of the growing threat posed by these pathogens has led WHO to classify carbapenem-resistant *K. pneumoniae* and *A. baumannii* as critical priority pathogens requiring urgent development of new therapeutic strategies (WHO, 2024). Similarly, the United States Centers for Disease Control and Prevention (CDC) categorizes carbapenem-resistant *A. baumannii* and carbapenem-resistant Enterobacterales among the most urgent antimicrobial resistance threats (CDC, 2019; Tamma et al., 2024). Despite ongoing efforts to develop novel antibiotics and antimicrobial combinations, the pace of resistance evolution continues to exceed antimicrobial discovery, emphasizing the urgent need for alternative strategies targeting biofilm formation, pathogen persistence, and device-associated colonization (Tamma et al., 2024).

## 4 Antimicrobial resistance mechanisms in *Klebsiella pneumoniae* and *Acinetobacter baumannii*

**Figure 2:**
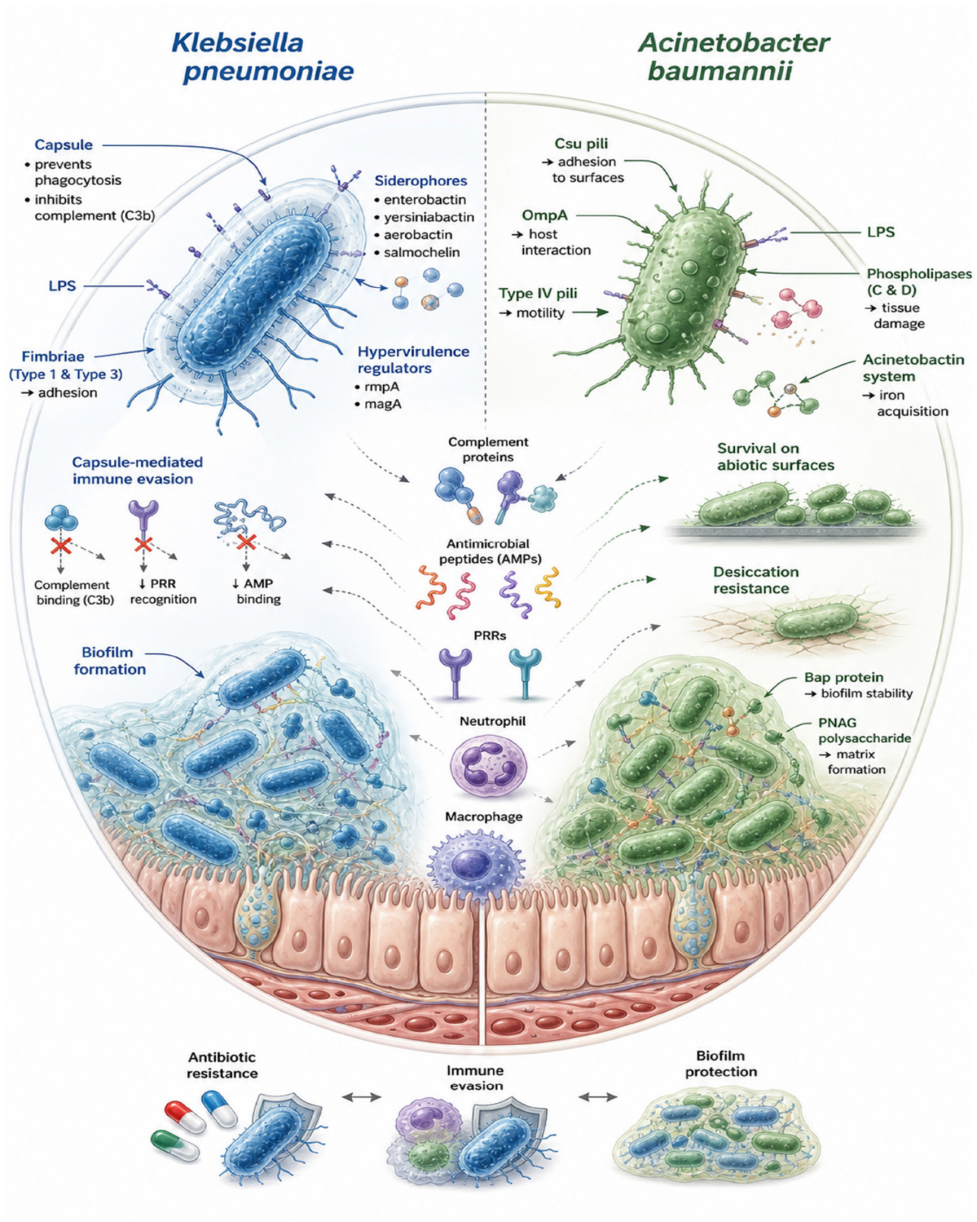
An overview of the major virulence determinants and antimicrobial resistance mechanisms of K. pneumoniae and A. baumannii, illustrating their contributions to persistence, immune evasion and therapeutic challenges in MDR-VAP. **Note**: LPS-lipopolysaccharide; PRRs- pattern recognition receptors; PNAG- poly-β-(1 → 6)-N-acetylglucosamine.

The increasing prevalence of MDR *K. pneumoniae* and *A. baumannii* in VAP is driven by the accumulation of multiple AMR mechanisms that collectively compromise the efficacy of several antibiotic classes (Yang et al., 2023; Salawudeen et al., 2023). In prolonged antibiotic exposure, invasive ventilation, and biofilm-associated growth further accelerate selection and dissemination of resistant clones, particularly among CRKP and CRAB variants, which are now considered critical priority pathogens by the WHO (WHO, 2024). The molecular determinants contributing to resistance, persistence, immune evasion, and biofilm formation in both pathogens are summarized in Figure 2.

### 4.1 Enzymatic inactivation of antibiotics

Enzymatic degradation of antibiotics, particularly through β-lactamase production, represents the most clinically significant resistance mechanism in both *K. pneumoniae* and *A. baumannii* (Bush and Bradford, 2020; Lawrence et al., 2024). β-lactamases hydrolyse the β-lactam ring present in penicillins, cephalosporins, monobactams, and carbapenems, thereby rendering these antibiotics ineffective. Based on the Ambler molecular classification system, β-lactamases are grouped into classes A, B, C, and D. Classes A, C, and D possess serine residues within their catalytic sites, whereas class B enzymes comprise metallo-β-lactamases (MBLs) that require divalent metal ions such as Zn^2+^ for catalytic activity (Bush and Bradford, 2020). ESBLs including Cefotaximase-Munich (CTX-M), Sulfhydryl reagent variable type (SHV) and Temoniera type (TEM) Lactamase enzymes, are widely disseminated among *K. pneumoniae* clinical isolates and confer resistance to third-generation cephalosporins and aztreonam (Yang et al., 2023). Carbapenem resistance in *K. pneumoniae* is primarily mediated by carbapenemases such as KPC, NDM, VIM, and OXA-48-like enzymes, many of which are encoded on mobile genetic elements that facilitate rapid horizontal dissemination (Salawudeen et al., 2023; Lawrence et al., 2024). KPC enzymes hydrolyse most β-lactams including carbapenems, whereas NDM enzymes confer resistance to carbapenems and cephalosporins but typically spare monobactams. OXA-48-like enzymes preferentially hydrolyse penicillins and carbapenems while demonstrating relatively weak activity against cephalosporins (Bush and Bradford, 2020).

In *A. baumannii*, carbapenem resistance is dominated by OXA-type carbapenemases including OXA-23, OXA-24/40, OXA-58, and OXA-143 families, which are strongly associated with nosocomial outbreaks and endemic ICU transmission (Hamidian and Nigro, 2019). Metallo-β-lactamases such as NDM, IMP, and VIM are less common but confer high-level resistance when present. Additionally, intrinsic AmpC cephalosporinases contribute to reduced susceptibility against broad-spectrum cephalosporins and frequently coexist with carbapenemases, further narrowing treatment options (Yang et al., 2023). The clinical consequences of β-lactamase-mediated resistance are profound. Severe infections caused by ESBL-producing strains often require treatment with carbapenems, whereas CRKP and CRAB infections frequently leave only limited therapeutic options including polymyxins, tigecycline, aminoglycosides, or combination regimens (Mantzana et al., 2023). Although, newer β-lactam/β-lactamase inhibitor combinations such as ceftazidime-avibactam demonstrate strong activity against KPC-producing isolates, they remain ineffective against most metallo-β-lactamases, significantly limiting their utility in NDM-producing strains (Mackow and van Duin, 2024).

### 4.2 Efflux pump-mediated resistance

Efflux pumps contribute substantially to MDR by actively exporting antibiotics and toxic compounds from bacterial cells, thereby lowering intracellular antimicrobial concentrations below therapeutic levels (Voidazan et al., 2020). In Gram-negative pathogens, many clinically important efflux systems belong to the resistance-nodulation-division (RND) family and function as tripartite complexes spanning the inner membrane, periplasm, and outer membrane. In *K. pneumoniae*, the AcrAB-TolC system is one of the best-characterized multidrug efflux pumps and contributes to reduced susceptibility against fluoroquinolones, tetracyclines, β-lactams, and chloramphenicol (Li et al., 2019). The plasmid-mediated OqxAB pump additionally contributes to resistance against quinolones, chloramphenicol, tigecycline, and disinfectants, supporting both antimicrobial resistance and environmental persistence in healthcare settings (Asokan et al., 2025).

Efflux-mediated resistance is particularly important in *A. baumannii*, where the RND-family multidrug efflux pumps *adeABC, adeIJK, and adeFGH-* each comprising a membrane fusion protein (*adeA, adeI and adeF*), an inner-membrane RND multidrug transporter (*adeB, adeJ and adeG*), and an outer membrane factor/channel (*adeC, adeK and adeH*), respectively contribute to broad-spectrum resistance phenotypes (Hamidian and Nigro, 2019). *AdeABC* primarily mediates resistance to aminoglycosides, fluoroquinolones, tetracyclines, tigecycline, and selected β-lactams, whereas *AdeIJK* contributes to intrinsic multidrug resistance and bacterial fitness, and *AdeFGH* has been linked to resistance to fluoroquinolones, chloramphenicol, trimethoprim, and biofilm formation (Hamidian and Nigro, 2019). Among these, AdeABC is the most extensively studied and is associated with reduced susceptibility to aminoglycosides, fluoroquinolones, tetracyclines, tigecycline, and certain β-lactams. Overexpression of AdeABC, often mediated through mutations in the AdeRS regulatory system, has also been associated with enhanced biofilm formation and increased tolerance to disinfectants and environmental stress (Abdi et al., 2020; Xie et al., 2025). Importantly, efflux systems frequently act synergistically with β-lactamase production and reduced membrane permeability, producing additive resistance effects that are difficult to overcome clinically. Although, numerous laboratory studies have confirmed the contribution of efflux pumps to MDR, their relative impact varies considerably among clinical isolates and infection settings (Hamidian and Nigro, 2019; Voidazan et al., 2020). Furthermore, the absence of clinically approved efflux pump inhibitors emphasizes the translational gap between mechanistic discoveries and effective therapeutic interventions.

### 4.3 Reduced outer membrane permeability

Reduced outer membrane permeability contributes to resistance by limiting antibiotic entry into bacterial cells. In Gram-negative pathogens, porins function as outer membrane channels that facilitate passive diffusion of hydrophilic molecules, including many β-lactam antibiotics (Voidazan et al., 2020). Alterations in porin expression or structure, therefore, significantly affect antimicrobial susceptibility.

In *K. pneumoniae*, OmpK35 and OmpK36 are the principal porins involved in uptake of cephalosporins and carbapenems. Reduced expression or structural modification of these proteins decreases membrane permeability and contributes to elevated resistance levels, particularly when combined with carbapenemase production (Alves et al., 2023). Mutations affecting loop regions within OmpK36 may narrow pore diameter and substantially impair carbapenem influx, increasing minimum inhibitory concentrations beyond therapeutic thresholds (Yang et al., 2023).

Similarly, reduced permeability contributes significantly to carbapenem resistance in *A. baumannii*. The outer membrane protein CarO is associated with uptake of imipenem and other carbapenems, and loss or modification of CarO is strongly associated with decreased carbapenem susceptibility (Hamidian and Nigro, 2019). Additional outer membrane proteins, including OmpA, contribute not only to reduced permeability but also to biofilm formation, adhesion, and immune evasion (Roy et al., 2022; Scribano et al., 2024). Environmental stress and antibiotic exposure may further modulate porin expression through global regulatory systems, enabling rapid adaptation to antimicrobial pressure (Yang et al., 2023).

### 4.4 Target-site modification

Modification of antibiotic targets represents another important mechanism underlying resistance in both pathogens. These alterations reduce antibiotic binding affinity while preserving essential bacterial cellular functions (Yang et al., 2023). Fluoroquinolone resistance commonly results from mutations within quinolone resistance-determining regions (QRDRs) of gyrA and parC, which encode DNA gyrase and topoisomerase IV, respectively (Hamidian and Nigro, 2019; Yang et al., 2023). These mutations reduce fluoroquinolone binding and contribute to high-level resistance.

Alterations in penicillin-binding proteins (PBPs) also contribute to β-lactam resistance. In *A. baumannii*, modified PBP2 has been associated with carbapenem resistance, particularly when combined with OXA-type carbapenemases and porin loss (Gedefie et al., 2023). Resistance to aminoglycosides may occur through methylation of 16S ribosomal RNA mediated by genes such as armA and rmtB, which interfere with antibiotic binding to the bacterial ribosome (Roy et al., 2022). Polymyxin resistance, increasingly reported among CRKP and CRAB isolates, commonly arises through modification of lipid A within the lipopolysaccharide (LPS) layer. In *K. pneumoniae*, this may involve plasmid-mediated *mcr* genes or mutations affecting regulatory systems such as *mgrB* and *pmrAB*. In *A. baumannii*, mutations in *pmrA*/*pmrB* and lipid A biosynthesis genes including *lpxA, lpxC*, and *lpxD* reduce polymyxin binding and contribute to colistin resistance (Gedefie et al., 2023; Alves et al., 2023).

### 4.5 Horizontal gene transfer and genetic plasticity

The remarkable adaptability of *K. pneumoniae* and *A. baumannii* is strongly linked to their capacity for horizontal gene transfer (HGT), which enables rapid acquisition and dissemination of resistance determinants within hospital environments (Annunziato, 2019). Resistance genes encoding carbapenemases, ESBLs, aminoglycoside-modifying enzymes, and efflux systems are frequently located on plasmids, integrons, and transposons that facilitate transfer between strains and bacterial species (Bollen et al., 2023).

In ICU settings, selective antibiotic pressure, prolonged hospitalization, and high-density microbial populations create favorable conditions for dissemination of resistant clones. Biofilm-associated growth further enhances HGT by increasing bacterial proximity and promoting plasmid exchange through conjugation (Bollen et al., 2023). Consequently, biofilms formed on ETTs and other medical devices act not only as reservoirs for persistent infection but also as environments that accelerate evolution and dissemination of antimicrobial resistance.

The convergence of enzymatic inactivation, efflux-mediated resistance, membrane impermeability, target-site modification, and horizontal gene transfer has therefore enabled *K. pneumoniae* and *A. baumannii* to emerge as highly adaptable and persistent ICU pathogens. These resistance mechanisms are further reinforced by biofilm-associated growth and immune evasion strategies, which collectively contribute to chronic device-associated infections and severe therapeutic failure in VAP (Bollen et al., 2023).

Although, the molecular mechanisms underlying antimicrobial resistance in *K. pneumoniae* and *A. baumannii* have been extensively characterized, much of the available evidence is derived from genomic analyses, *in vitro* susceptibility testing, and experimental laboratory models. While these studies have substantially improved understanding of resistance evolution, their direct translation to clinical management remains challenging because resistance phenotypes are influenced by host factors, biofilm-associated physiology, antimicrobial exposure, and polymicrobial interactions that are not fully reproduced under laboratory conditions. Furthermore, resistance mechanisms frequently coexist within individual clinical isolates, complicating efforts to attribute therapeutic failure to any single determinant. Future studies integrating genomic, transcriptomic, and clinical outcome data are needed to better define the relative contribution of individual resistance mechanisms to treatment failure in VAP.

## 5 Biofilm formation and persistence in *Klebsiella pneumoniae* and *Acinetobacter baumannii*

**Figure 3:**
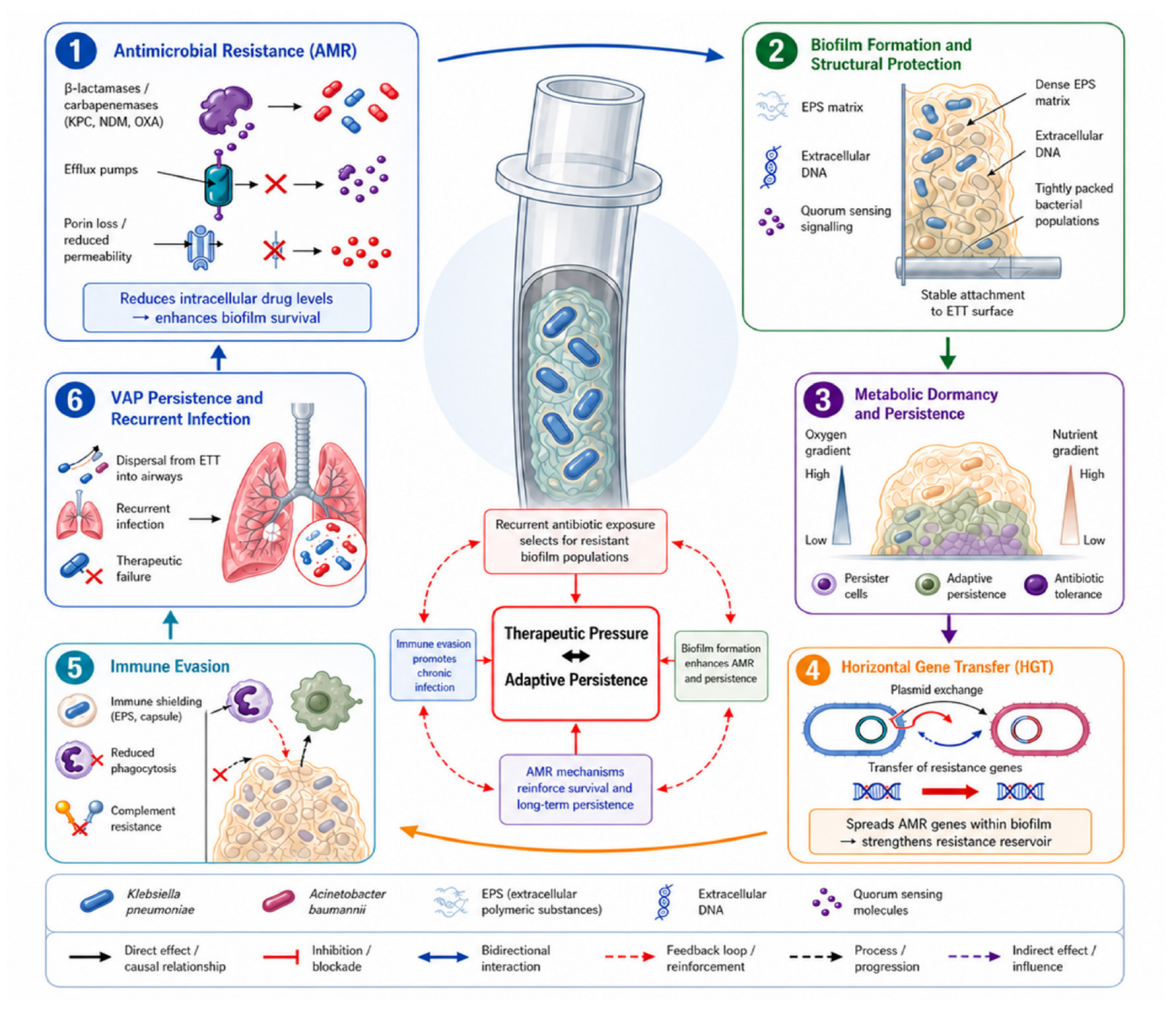
Antimicrobial resistance, biofilm formation, and host-pathogen interactions contributes toward persistence and therapeutic failure in MDR-VAP.

The persistence of *K. pneumoniae* and *A. baumannii* in VAP is strongly linked to their capacity to form highly structured biofilms on ETTs and other abiotic surfaces within ICU environments (Marcut et al., 2023; Cavallo et al., 2023). Biofilms are organized microbial communities embedded within a self-produced EPS matrix composed of polysaccharides, proteins, lipids, and extracellular DNA (eDNA), which collectively enhance bacterial survival under antimicrobial and host immune pressure (Deshmukh-Reeves et al., 2025; Flemming et al., 2025). In mechanically ventilated patients, humidified airflow, respiratory secretions, and host-derived proteins create favorable conditions for bacterial adhesion and biofilm maturation on ETT surfaces, allowing pathogens to persist as reservoirs for recurrent lower respiratory tract infection (Marcut et al., 2023). Periodic dispersal of planktonic cells or biofilm fragments from colonized ETTs contributes directly to VAP pathogenesis and recurrent infection episodes (Mishra et al., 2024). The interconnected mechanisms underlying biofilm-mediated persistence, antimicrobial tolerance, immune evasion, and recurrent infection in MDR-VAP infections are summarized in Figure 3.

### 5.1 Initial adhesion and biofilm development

Biofilm formation begins with initial bacterial attachment to abiotic surfaces, followed by microcolony formation and progressive EPS accumulation. In *K. pneumoniae*, fimbrial adhesins are central mediators of surface attachment and colonization (Ashwath et al., 2022). Type 1 fimbriae facilitate adhesion to epithelial cells and contribute to colonization of host tissues and indwelling medical devices, whereas type 3 fimbriae encoded by the mrkABCDF operon are particularly important for attachment to abiotic materials such as plastics and silicone surfaces commonly found in ETTs (Gato et al., 2020; Ashwath et al., 2022). Strains lacking the *mrk* operon exhibit significantly impaired biofilm formation under flow conditions, highlighting the importance of type 3 fimbriae during early biofilm development (Gato et al., 2020). Additional adhesion systems, including ECP and Kpf fimbrial clusters, further contribute to epithelial attachment and biofilm stability (Gato et al., 2020).

Capsular polysaccharides also influence biofilm development in *K. pneumoniae*. Although, the capsule enhances protection against phagocytosis and complement-mediated killing, excessive capsule production may impair adhesion by masking surface-associated adhesins (Li et al., 2023). Hyper-mucoviscous strains carrying regulators such as *rmpA* and *magA* often display enhanced invasiveness but reduced surface attachment, suggesting that successful biofilm formation requires a balance between capsule expression and fimbrial activity (Buffet et al., 2021; Yao et al., 2025).

In *A. baumannii*, initial adhesion is mediated primarily by Csu pili, outer membrane protein A (OmpA), and type IV pili (Manjusha et al., 2023; Ahmad et al., 2023). The Csu chaperone-usher pili assembly system promotes attachment to abiotic surfaces including ETTs, catheters, and ventilator components, while the BfmRS two-component regulatory system controls expression of adhesion-associated genes required for microcolony formation (Zavialov et al., 2025). OmpA contributes not only to adhesion and epithelial interaction but also to intercellular stabilization within developing biofilms through interaction with extracellular DNA (Elkheloui et al., 2020; Ranjbar et al., 2022). Type IV pili additionally support twitching motility and surface colonization, facilitating expansion of biofilm communities across abiotic surfaces (Ahmad et al., 2023).

### 5.2 Biofilm maturation and regulatory networks

Following attachment, biofilms undergo maturation through coordinated production of extracellular matrix components and activation of regulatory signaling pathways. In *A. baumannii*, the biofilm-associated protein (Bap) and the exopolysaccharide poly-β-(1,6)-N-acetylglucosamine (PNAG), synthesized by the pgaABCD locus, are major structural components of the mature biofilm matrix (Elkheloui et al., 2020; Upmanyu et al., 2024). Bap promotes intercellular aggregation and formation of complex three-dimensional biofilm architecture, whereas PNAG contributes to matrix stability and surface adherence. Mutants deficient in Bap or PNAG production exhibit significantly weaker biofilm formation and impaired structural integrity (Ranjbar et al., 2022).

In both pathogens, extracellular DNA further stabilizes the EPS matrix and enhances interbacterial cohesion within mature biofilms (Upmanyu et al., 2022). Iron acquisition systems also contribute significantly to biofilm maturation under iron-restricted host conditions. *K. pneumoniae* produces siderophores including enterobactin, aerobactin, yersiniabactin, and salmochelin, which support bacterial growth and biofilm robustness in nutrient-limited environments (Choby et al., 2020; Guerra et al., 2022). Similarly, *A. baumannii* utilizes the acinetobactin system encoded by the *bas*/*bau* gene clusters for iron acquisition, and acinetobactin-deficient mutants demonstrate reduced biofilm formation on abiotic surfaces (Cook-Libin et al., 2022; Jeong et al., 2024).

Biofilm maturation is tightly coordinated through quorum sensing and intracellular signaling networks. *K. pneumoniae* primarily utilizes the LuxS/AI-2 quorum sensing system, whereas *A. baumannii* employs the AbaI/AbaR signaling circuit to regulate adhesion, EPS production, and biofilm maturation in response to population density (Roy et al., 2022; Venkateswaran et al., 2023). Cyclic-di-GMP signaling additionally regulates the transition from planktonic growth to sessile biofilm formation by promoting expression of adhesins and matrix-associated genes (Berry et al., 2024). In *A. baumannii*, the BfmRS regulatory system coordinates expression of pili and biofilm-associated proteins in response to environmental stress, thereby enhancing persistence under ICU-associated conditions (Niu et al., 2024; Zavialov et al., 2025).

### 5.3 Biofilm-mediated persistence and antimicrobial tolerance

Biofilm-associated growth profoundly alters bacterial physiology and contributes substantially to antimicrobial tolerance and chronic persistence (Sharif and Yadav, 2025). The EPS matrix restricts penetration of antimicrobial agents, particularly hydrophilic antibiotics, creating concentration gradients that expose deeper biofilm layers to sub-inhibitory drug concentrations. This environment promotes survival of tolerant bacterial subpopulations and facilitates selection of resistant phenotypes during antimicrobial exposure (Niu et al., 2024).

Within mature biofilms, bacterial cells frequently adopt metabolically inactive or slow-growing states that reduce susceptibility to antibiotics targeting active cellular processes (Niu et al., 2024). Persister cells, regulated in part through stringent response pathways and toxin-antitoxin systems, exhibit transient antibiotic tolerance and can repopulate biofilms following treatment cessation (Kunnath et al., 2024; Niu et al., 2024). Efflux systems such as OqxAB in *K. pneumoniae* and AdeABC in *A. baumannii* further enhance persistence by reducing intracellular antimicrobial accumulation and supporting adaptation to environmental stress (Abdi et al., 2020; Asokan et al., 2025).

Biofilms also promote immune evasion by limiting complement deposition, reducing phagocytosis, and restricting penetration of antimicrobial peptides and antibodies into deeper biofilm layers (Talyansky et al., 2021; Dijksteel et al., 2021). In *K. pneumoniae*, capsular polysaccharides inhibit complement-mediated opsonization and reduce effective recognition by host immune cells, while in *A. baumannii*, OmpA, capsular structures, and matrix-associated components interfere with complement activation and opsonophagocytic clearance (Scribano et al., 2024). These mechanisms collectively contribute to persistence within the respiratory tract and support recurrent device-associated infections.

Biofilms also facilitate HGT, further promoting the dissemination of antimicrobial resistance determinants and persistence within ICU settings (Bollen et al., 2023). Therefore, biofilms formed on ETTs act not only as reservoirs for persistent infection but also as environments that accelerate the evolution and spread of antimicrobial resistance within ICU settings (Bollen et al., 2023). Considerable experimental evidence supports the role of biofilms in antimicrobial tolerance and persistent infection. However, most mechanistic studies have been performed using static *in vitro* biofilm models that incompletely replicate the complex physiological conditions of the ventilated airway. Biofilm composition, oxygen gradients, host immune interactions, and polymicrobial communities differ substantially *in vivo*, limiting extrapolation of laboratory findings to clinical practice. Consequently, despite strong biological possibility, the precise contribution of biofilm disruption to improved patient outcomes remains insufficiently established through randomized clinical trials.

Accurate detection and monitoring of biofilms remain major challenges in the clinical management of VAP because conventional microbiological cultures primarily identify planktonic bacteria and provide limited information regarding biofilm-associated infections (Yang et al., 2025). Scanning electron microscopy (SEM), confocal laser scanning microscopy (CLSM), fluorescence *in situ* hybridization (FISH), and molecular techniques such as quantitative PCR and metagenomic sequencing have been widely used in research settings to characterize biofilm architecture, microbial composition, and resistance determinants on ETTs (Sinha et al., 2025; Rothmaier et al., 2026). More recently, advanced imaging approaches, biosensors, and AI-assisted image analysis have shown promise for improving biofilm detection and monitoring. However, most of these technologies remain confined to experimental or laboratory settings because of their cost, technical complexity, and limited clinical validation. Consequently, there remains an unmet need for rapid, standardized, and clinically applicable methods for detecting biofilms and monitoring treatment response in patients with MDR-VAP (Yang et al., 2025; Rothmaier et al., 2026).

## 6 Host-pathogen interactions in VAP

The respiratory tract possesses multiple innate defense mechanisms that normally prevent microbial colonization and lower airway infection, including mucociliary clearance, antimicrobial peptides, complement activation, alveolar macrophages, and neutrophil-mediated phagocytosis (Dijksteel et al., 2021). In mechanically ventilated patients, however, disruption of airway integrity by ETTs, impaired mucociliary clearance, prolonged antibiotic exposure, and critical illness compromise these protective barriers and facilitate colonization by opportunistic MDR pathogens (Marcut et al., 2023). Among these, *K. pneumoniae* and *A. baumannii* possess diverse virulence and immune evasion strategies that enable persistent survival within the respiratory tract despite antimicrobial therapy and host immune responses.

A major immune evasion mechanism in *K. pneumoniae* is the production of capsular polysaccharides, which inhibit complement activation and reduce opsonophagocytic clearance by neutrophils and macrophages (Choby et al., 2020). The capsule interferes with deposition of complement component C3b, a key opsonin that promotes phagocytic recognition and clearance of bacteria, thereby impairing effective uptake by neutrophils and macrophages (Talyansky et al., 2021). Hypermucoviscous strains expressing regulators such as *rmpA* and *magA* exhibit enhanced resistance to serum killing and increased persistence within host tissues (Buffet et al., 2021). Additionally, siderophore systems including enterobactin, aerobactin, and yersiniabactin support bacterial survival under iron-restricted host conditions and contribute indirectly to persistence and virulence during pulmonary infection (Choby et al., 2020; Guerra et al., 2022).

Similarly, *A. baumannii* employs multiple mechanisms to evade innate immune clearance. Outer membrane protein A (OmpA) contributes to epithelial adhesion, biofilm stabilization, and modulation of host immune responses, while capsular structures and LPS modifications reduce complement-mediated killing and antimicrobial peptide activity (Roy et al., 2022; Scribano et al., 2024). The pathogen also exhibits remarkable tolerance to oxidative stress and desiccation, allowing prolonged persistence on abiotic ICU surfaces and facilitating recurrent transmission in healthcare environments (Manjusha et al., 2023). In addition, phospholipases and other secreted virulence factors contribute to epithelial damage and inflammatory dysregulation during severe pulmonary infection (Elkheloui et al., 2020).

Biofilm-associated growth further amplifies immune evasion in both pathogens. The EPS matrix acts as a physical barrier that limits penetration of complement proteins, antimicrobial peptides, and phagocytic immune cells into deeper biofilm layers (Dijksteel et al., 2021). Biofilms also impair neutrophil migration and reduce effective bacterial clearance by promoting localized immune dysregulation and chronic inflammation (Sharif and Yadav, 2025). Within mature biofilms, metabolically dormant persister cells exhibit reduced immunogenicity and enhanced tolerance to oxidative and antimicrobial stress, enabling prolonged survival despite ongoing host immune responses (Kunnath et al., 2024). Therefore, biofilm-associated colonization of ETTs serves as a persistent reservoir for recurrent bacterial dispersal into the lower respiratory tract, contributing to chronic infection, recurrent VAP, and therapeutic failure (Mishra et al., 2024). The major immune evasion strategies employed by *K. pneumoniae* and *A. baumannii*, together with their implications for persistence and current therapeutic challenges, are summarized in Table 1.

**Table 1:** Comparative immune evasion mechanisms of *Klebsiella pneumoniae* and *Acinetobacter baumannii* and their contribution to persistence in ventilator-associated pneumonia.

Immune evasion strategy	*K. pneumoniae*	*A. baumannii*	Impact on persistence	Current challenges	References
Capsule-mediated complement resistance	Capsule inhibits C3b deposition and limits membrane attack complex formation	Capsule and OmpA interfere with complement-mediated killing	Reduced opsonization and phagocytic clearance promotes prolonged airway colonization	Capsule diversity complicates vaccine and monoclonal antibody development, mechanisms linking capsule composition to treatment outcomes remain incompletely understood	Talyansky et al., 2021; Jayaraman et al., 2024
Biofilm-associated immune shielding	Capsule and EPS matrix reduce immune recognition and antimicrobial peptide access	PNAG, Bap, and EPS-mediated protection restrict immune penetration	Promotes persistent ETT colonization, recurrent VAP, and reduced antimicrobial efficacy	Limited clinically approved anti-biofilm therapies; uncertainty regarding optimal biofilm-disrupting interventions in VAP	Fu et al., 2023; Mantovani and Garlanda, 2023; Voronko et al., 2025
Impaired surfactant-mediated clearance	Capsular polysaccharides reduce binding of surfactant proteins	Biofilm matrix interferes with SP-A/SP-D-mediated opsonization	Reduces bacterial agglutination and clearance from the respiratory tract	Role of surfactant-targeted therapies in MDR-VAP remains largely unexplored	Ma and McClean, 2021; Mantovani and Garlanda, 2023
Modulation of innate immune recognition	Capsule masks pathogen-associated molecular patterns and reduces TLR4 stimulation	Lipid A modification dampens TLR4-mediated inflammatory signaling	Delays effective immune activation and facilitates persistent colonization	Host-pathogen signaling pathways during chronic biofilm-associated VAP remain poorly characterized	Skrzypczak-Wiercioch and Sałat, 2022
Inflammasome evasion and immune modulation	Capsule and biofilm reduce exposure to inflammasome-mediated responses	OmpA modulates inflammasome activation while biofilms protect against immune clearance	Contributes to chronic infection and ineffective bacterial eradication	Precise role of inflammasome signaling in MDR-VAP progression requires further investigation	Blevins et al., 2022; Dai et al., 2022
Evasion of cytosolic nucleic acid sensing pathways	Intracellular persistence mechanisms may reduce nucleic acid detection	DNA modifications may reduce AIM2-mediated sensing, STING responses remain variable	Weakens downstream interferon-mediated antibacterial responses	Significance of AIM2/STING pathways in VAP remains incompletely defined	Fan et al., 2023; Zhang et al., 2024
Resistance to antimicrobial peptides (AMPs)	Capsule and biofilm matrix sequester defensins and cathelicidins	Biofilm matrix and membrane modifications reduce AMP binding and penetration	Enhances survival despite innate antimicrobial defenses	Translation of AMP-based therapies into clinical VAP management remains limited	Fu et al., 2023; Voronko et al., 2025
Environmental persistence and abiotic surface adaptation	Colonizes respiratory devices and hospital equipment following barrier disruption	Exceptional desiccation tolerance facilitates prolonged survival on ICU surfaces	Sustains transmission, recolonization, and recurrent infection	Strategies preventing recolonization of ventilator equipment remain insufficiently optimized	Roy et al., 2022; Bustos et al., 2025
Combined AMR-biofilm-immune evasion phenotype	Frequently observed in carbapenem-resistant and hypervirulent strains	Common among MDR/XDR hospital-adapted clones	Creates a self-reinforcing cycle of persistence, recurrent infection, and therapeutic failure	Need for integrated therapeutic approaches targeting resistance, biofilms, and immune evasion simultaneously	Talyansky et al., 2021; Roy et al., 2022; Fu et al., 2023

Although virulence factors, antimicrobial resistance, and biofilm formation each contribute to the pathogenesis of MDR-VAP, they influence disease progression through distinct mechanisms. Virulence factors primarily promote colonization, immune evasion, and host tissue damage (Jayaraman et al., 2024), whereas antimicrobial resistance reduces the efficacy of conventional antibiotics. In contrast, biofilm formation facilitates bacterial persistence by limiting antimicrobial penetration, promoting persister-cell formation, and enhancing horizontal gene transfer (Voronko et al., 2025). Therefore, emerging therapeutic strategies must target multiple pathogenic processes simultaneously to improve treatment outcomes.

## 7 Conventional therapeutic approaches and their limitations

The management of VAP remains a major therapeutic challenge due to the convergence of extensive antimicrobial resistance, biofilm-associated persistence, and impaired antibiotic penetration within the infected respiratory tract. Although, advances in antimicrobial development have expanded treatment options for carbapenem-resistant Gram-negative pathogens, clinical outcomes remain suboptimal, particularly in critically ill patients with established biofilm-associated infections. Mortality associated with MDR-VAP frequently exceeds 30%−50%, with rates often surpassing 60% in patients infected with CRAB, highlighting the urgent need for effective therapeutic strategies (Torres et al., 2025; Tamma et al., 2024).

As described in Figures 2 and 3, the interconnected processes of antimicrobial resistance, biofilm formation, and host-pathogen interactions provide multiple therapeutic targets, forming the basis for the emerging anti-biofilm strategies. Current treatment recommendations are primarily guided by pathogen identification, resistance mechanisms, local epidemiology, antimicrobial susceptibility testing, and contemporary international guidelines, including those from the American Thoracic Society/Infectious Diseases Society of America (ATS/IDSA) and the European Society of Clinical Microbiology and Infectious Diseases (ESCMID), which emphasize prompt administration of appropriate empirical therapy, subsequent de-escalation based on microbiological results, and antimicrobial stewardship (Scudeller et al., 2025; Venkatesan, 2026; St. Peter et al., 2026; Elshenawy, 2026). Biofilm-associated bacteria exhibit marked phenotypic tolerance that substantially reduces antibiotic efficacy despite *in vitro* susceptibility (Sharma et al., 2019). Furthermore, biofilms are implicated in approximately 65% of microbial infections and more than 80% of chronic infections, emphasizing their importance as major contributors to therapeutic failure and recurrent disease (Ahmad et al., 2023).

### 7.1 Device-based strategies to prevent biofilm formation

Given the central role of ETT colonization in the pathogenesis of VAP, considerable efforts have focused on developing device-based interventions that prevent bacterial attachment and biofilm establishment before mature and treatment-refractory communities develop (Wang et al., 2024). Unlike systemic antimicrobial therapies, which are often limited by poor biofilm penetration and the emergence of antimicrobial resistance, ETT-based strategies target the earliest stages of colonization and may therefore reduce both biofilm burden and subsequent infection risk (Chen et al., 2022). Current approaches include antimicrobial-coated ETTs, noble metal alloy coatings, subglottic secretion drainage (SSD), and anti-adhesive surface modifications, all of which aim to interrupt key steps in biofilm formation (Sanaie et al., 2022).

Among these, silver-coated ETTs remain the most extensively studied and clinically validated anti-biofilm devices. Silver exerts broad-spectrum antimicrobial activity through the sustained release of Ag^+^ ions, which disrupt bacterial membrane integrity, interfere with thiol-containing enzymes, impair DNA replication, and induce oxidative stress (Chen et al., 2022; Yang et al., 2024). These multimodal effects are particularly effective against MDR pathogens because they reduce the likelihood of resistance development compared with conventional antibiotics (Chen et al., 2022). Clinical evidence supporting silver-coated ETTs was established by the North American Silver-Coated ETTs (NASCENT) randomized trial, which demonstrated a 36% reduction in microbiologically confirmed VAP and a significant delay in infection onset among mechanically ventilated patients receiving silver-coated tubes compared with standard ETTs (Yang et al., 2024). Subsequent investigations reported VAP incidences of approximately 3.5% in patients intubated with silver-coated ETTs compared with 6.7% in those receiving conventional devices, supporting their ability to suppress early bacterial colonization and biofilm development (Chen et al., 2022). However, silver coatings do not completely prevent biofilm formation during prolonged ventilation, and concerns regarding coating durability, cost, and gradual depletion of active silver ions remain important limitations (Yang et al., 2024). While silver-coated ETTs and subglottic secretion drainage have demonstrated clinical benefit, most other emerging anti-biofilm strategies remain supported primarily by preclinical evidence and require validation in well-designed clinical trials (Yang et al., 2024).

To improve long-term antimicrobial performance, noble metal alloy (NMA) coatings incorporating combinations of silver, gold, and palladium have been developed (Yang et al., 2024). These coatings provide sustained antimicrobial activity while minimizing corrosion and maintaining surface stability over extended periods. In a randomized clinical trial involving mechanically ventilated ICU patients, NMA-coated ETTs reduced VAP incidence from 43.2% to 27.8% (*p* = 0.03) compared with standard tubes, while also reducing bacterial colonization and antibiotic consumption (Marcut et al., 2023). Similar findings have been reported in studies evaluating the BIP ETT, where coated devices delayed VAP onset and lowered microbial burden within respiratory secretions. Although, clinical data remain more limited than those available for silver-coated ETTs, these findings suggest that sustained-release metallic coatings may provide durable protection against biofilm establishment in high-risk patients (Damas et al., 2022).

An alternative approach focuses on reducing the microbial inoculum available for biofilm formation rather than directly targeting bacterial viability. Secretions accumulating above the ETT cuff provide a nutrient-rich reservoir that facilitates microbial growth and repeated microaspiration into the lower respiratory tract (Sanaie et al., 2022). SSD-equipped ETTs continuously remove these secretions, thereby limiting bacterial access to the airway and reducing opportunities for biofilm development. Meta-analyses have demonstrated that SSD can reduce VAP incidence by up to 45%, while also delaying infection onset and shortening the duration of mechanical ventilation (Sanaie et al., 2022). Consequently, SSD has become one of the few device-based interventions incorporated into major VAP prevention guidelines and is frequently combined with other anti-biofilm technologies.

More recently, attention has shifted toward anti-adhesive surface modifications that prevent bacterial attachment without relying on antimicrobial release. Among these, micropatterned surfaces inspired by shark skin topography have shown particular promise (Chen et al., 2022). The Sharklet^®^ design generates a micro-structured surface that physically impedes bacterial settlement and biofilm development. Experimental studies demonstrated greater than 70% reductions in *P. aeruginosa* biofilm formation compared with unmodified surfaces (Marcut et al., 2023). As these approaches do not depend on bactericidal activity, they exert minimal selective pressure for resistance development and may provide a complementary strategy to antimicrobial coatings (Marcut et al., 2023).

Therefore, device-based interventions represent one of the most clinically advanced approaches for preventing biofilm-associated VAP. While antimicrobial coatings, secretion drainage systems, and anti-adhesive surfaces differ mechanistically, all seek to disrupt the critical transition from initial bacterial attachment to mature biofilm formation (Chen et al., 2022). However, no currently available technology completely eliminates biofilm development during prolonged mechanical ventilation, highlighting the need for complementary therapeutic strategies capable of disrupting established biofilms and enhancing antimicrobial efficacy. These device-based interventions should be regarded as adjuncts to, rather than replacements for, guideline-recommended antimicrobial therapy and established VAP prevention bundles. Moreover, the clinical evidence supporting these interventions is heterogeneous, with differences in patient populations, study design, ventilation duration, and definitions of VAP limiting direct comparisons across studies. While silver-coated and subglottic secretion drainage ETTs have demonstrated clinical benefits, many other device-based approaches remain supported primarily by preclinical investigations and require further validation in multicenter randomized clinical trials. A summary of device-based VAP prevention strategies is listed in Table 2.

**Table 2:** Device-based strategies for preventing biofilm-associated VAP, including their mechanisms, reported efficacy, advantages and current limitations.

Strategy	Mechanism of action	Key findings	Advantages	Limitations	References
Silver-coated ETTs	Sustained Ag^+^ release causing membrane disruption, enzyme inhibition, DNA damage, and oxidative stress	NASCENT trial: 36% reduction in microbiologically confirmed VAP, delayed VAP onset. VAP incidence reduced from 6.7% to 3.5% in subsequent studies	Strongest clinical evidence, broad-spectrum activity, FDA-approved	Cost, gradual silver depletion, incomplete prevention of biofilm formation	Chen et al., 2022; Yang et al., 2024
Noble metal alloy (Ag-Au-Pd) ETTs	Controlled release of antimicrobial metal ions with enhanced coating stability	VAP incidence reduced from 43.2% to 27.8% (*p* = 0.03), reduced bacterial colonization and antibiotic use	Long-lasting antimicrobial activity, reduced corrosion	Limited large-scale clinical validation	Marcut et al., 2023; Damas et al., 2022
Subglottic secretion drainage (SSD) ETTs	Continuous removal of contaminated secretions above the cuff, reducing micro-aspiration	Up to 45% reduction in VAP incidence, delayed VAP onset, reduced duration of mechanical ventilation	Guideline-recommended, strong evidence base	Does not directly eradicate biofilms	Sanaie et al., 2022
Micropatterned (Sharklet^®^) surfaces	Physical inhibition of bacterial attachment through microtopography	>70% reduction in *P. aeruginosa* biofilm formation in experimental models	No antimicrobial release, minimal resistance pressure	Primarily preclinical evidence	Marcut et al., 2023
Hydrophilic/Zwitterionic coatings	Reduction of protein adsorption and bacterial attachment through surface hydration layers	Significant reduction in bacterial adhesion *in vitro*	Non-antibiotic approach, antifouling properties	Limited clinical validation	Kazemzadeh-Narbat et al., 2021

### 7.2 Emerging anti-biofilm therapeutics

The limited efficacy of conventional antimicrobial therapy against established biofilms has stimulated the development of therapeutic strategies that directly target biofilm architecture, bacterial persistence mechanisms, and intercellular communication pathways (Flemming et al., 2025). Unlike device-based preventive approaches that aim to inhibit initial colonization, these interventions seek to disrupt mature biofilms, enhance antimicrobial penetration, and restore susceptibility to host immune responses and antibiotic treatment. Although, most remain in preclinical development, emerging anti-biofilm therapeutics have demonstrated promising activity against MDR *K. pneumoniae* and *A. baumannii* and may provide valuable adjuncts to conventional antimicrobial therapy (Zhao et al., 2023).

Nanotechnology-based approaches have attracted considerable attention owing to their ability to simultaneously target multiple components of the biofilm microenvironment. Among these, silver nanoparticles (AgNPs) remain the most extensively investigated due to their broad-spectrum antimicrobial activity mediated through membrane disruption, protein denaturation, DNA damage, and reactive oxygen species (ROS) generation (Lethongkam et al., 2023). Unlike conventional antibiotics that target specific cellular pathways, AgNPs exert multifaceted antimicrobial effects, reducing the likelihood of resistance development. *In vitro* and experimental studies have demonstrated 99%−99.999% reductions in bacterial adhesion and colonization, while AgNP-incorporated coatings effectively inhibited biofilm formation by MDR Gram-negative pathogens, including *K. pneumoniae* and *A. baumannii* (Lethongkam et al., 2023). Similarly, antibiotic-loaded and nanoparticle-enhanced hydrogel systems have shown substantial anti-biofilm activity by combining anti-adhesive properties with sustained antimicrobial delivery. Several pre-clinical formulations achieved greater than 95% reductions in bacterial adhesion within 4 h and >99.99% reductions after 24 h, while maintaining antimicrobial activity for more than 30 days under experimental conditions (Wouters et al., 2024). These multifunctional systems are particularly useful during prolonged mechanical ventilation, where continuous suppression of biofilm development is required.

A second group of emerging therapies focuses on direct disruption of the biofilm matrix. As the EPS matrix acts as both a physical barrier and a protective niche for bacterial persistence, enzymatic degradation has emerged as a promising strategy to improve antimicrobial penetration and facilitate immune clearance (Yang et al., 2023). DNases, proteases, and glycosidases target key matrix components, including extracellular DNA and polysaccharides, thereby destabilizing biofilm structure. Notably, a recently identified bovine microbial glycosidase (GH-B2) inhibited *K. pneumoniae* biofilm formation (IC_50_ = 2.50 μM) and disrupted mature biofilms (EC_50_ = 1.94 μM) by degrading matrix polysaccharides, while enhancing meropenem efficacy and improving immune-cell-mediated clearance of biofilm-associated bacteria (Ramakrishnan et al., 2024). Similarly, nitric oxide (NO)-releasing systems have demonstrated the ability to induce biofilm dispersal through modulation of intracellular cyclic-di-GMP signaling while simultaneously exerting direct antimicrobial effects. In pre-clinical studies, S-nitroso-N-acetylpenicillamine (SNAP) based Nitrogen oxide releasing coatings achieved approximately 93% reductions in bacterial colonization within 24 h, highlighting their potential as adjunctive anti-biofilm therapies (Aveyard et al., 2024).

Antimicrobial peptides (AMPs) and related membrane-active compounds represent another promising class of biofilm-targeted therapeutics. In contrast to conventional antibiotics, AMPs rapidly disrupt bacterial membranes and can penetrate biofilm matrices, allowing activity against both metabolically active and dormant bacterial populations (Dijksteel et al., 2021). AMPs including defensins and cathelicidins, provide another important chemical barrier. These peptides are produced by epithelial cells and immune cells, and they disrupt bacterial membranes through electrostatic interactions that lead to pore formation and lysis (Voronko et al., 2025). LL-37, the only known human cathelicidin, exhibits direct activity against Gram-negative bacteria including *K. pneumoniae* and *A. baumannii*. Experimental studies have demonstrated that, AMP-functionalized systems have resulted in 90%−99% reductions in biofilm formation, while AMP-loaded nanoparticles disrupted approximately 80% of mature biofilms in experimental models (Atefyekta et al., 2019; Roque-Borda et al., 2021). Synthetic AMP mimics, such as ceragenins, have been developed to overcome the limited stability of natural peptides and exhibit broad-spectrum activity against MDR Gram-negative pathogens, including *K. pneumoniae* and *A. baumannii*. These agents combine potent antimicrobial activity with reduced susceptibility to enzymatic degradation (Roque-Borda et al., 2021).

In addition to directly targeting biofilm structure, anti-virulence strategies have emerged as a complementary approach to impair bacterial persistence. Quorum sensing (QS) regulates biofilm maturation, virulence factor production, and adaptive stress responses in many clinically important pathogens (Roy et al., 2022). Consequently, disruption of QS signaling may attenuate pathogenicity without exerting the selective pressures typically associated with bactericidal therapies. Several natural compounds, including resveratrol, thymol, and carvacrol, have demonstrated anti-biofilm activity through interference with bacterial communication pathways and stress responses (Niu et al., 2024). Although, generally insufficient as standalone therapies, these agents may enhance the activity of conventional antimicrobials. A recent study by Migliaccio et al. (2024) examined susceptibility of *A. baumannii* biofilms to chlorhexidine (CHX) and benzalkonium chloride (BZK). They found that extremely high concentrations were required to eradicate mature biofilms. CHX at up to 16 × its planktonic minimum inhibitory concentration (MIC; 512 mg/L in their strains) was needed to achieve a complete kill of *A. baumannii* biofilm, and even 8 × MIC (256 mg/L) only reduced biofilm biomass by 30% (Migliaccio et al., 2024). Despite promising preclinical results, most emerging anti-biofilm therapies remain limited by challenges related to toxicity, stability, manufacturing scalability, regulatory approval, and the lack of robust clinical validation. In addition, large-scale production, cost-effectiveness, quality control, and integration into existing clinical workflows and the availability of appropriate healthcare infrastructure remain important barriers to their widespread adoption, particularly in resource-limited healthcare settings (Lethongkam et al., 2023). Technologies such as silver-coated ETTs, nanoparticle-based coatings, and other advanced biomaterials must demonstrate not only clinical efficacy but also economic value and feasibility for implementation across diverse healthcare systems (Niu et al., 2024).

Although, many emerging anti-biofilm strategies are designed to reduce the selective pressure associated with conventional antibiotics, bacterial adaptation remains a potential concern (Lethongkam et al., 2023). Experimental studies have demonstrated that prolonged or repeated exposure to nanoparticles may induce adaptive stress responses, alterations in membrane composition, enhanced efflux pump activity, and increased biofilm formation in some bacterial species (Atefyekta et al., 2019) Similarly, resistance or reduced susceptibility to antimicrobial peptides may arise through modifications of membrane charge, proteolytic degradation, or activation of adaptive regulatory pathways (Atefyekta et al., 2019; Roque-Borda et al., 2021). While quorum-sensing inhibitors are generally considered less likely to drive resistance because they target bacterial communication rather than viability, adaptive mutations and compensatory regulatory mechanisms have been reported under experimental conditions (Lethongkam et al., 2023). Continued surveillance and careful evaluation of bacterial adaptation will be essential during the development and clinical implementation of emerging anti-biofilm therapies.

Addressing these translational challenges through standardized manufacturing processes, regulatory evaluation, and well-designed clinical trials will be essential before these technologies can be routinely incorporated into the management of MDR-VAP. In addition, successful clinical implementation of these technologies should align with antimicrobial stewardship principles by improving antibiotic efficacy, reducing unnecessary antimicrobial exposure, and potentially slowing the emergence of antimicrobial resistance, although robust clinical evidence is still required to support their integration into stewardship programs. Nevertheless, by targeting mechanisms that promote biofilm persistence, these approaches represent promising adjuncts to conventional antimicrobial therapy (Migliaccio et al., 2024).

### 7.3 Phage therapy and phage-derived depolymerases

Bacteriophage therapy is a promising strategy for the treatment of biofilm-associated MDR infections for its ability to specifically target bacterial pathogens while preserving the normal microbiota (MacNair et al., 2024). Lytic phages infect susceptible bacterial cells, replicate intracellularly, and induce bacterial lysis, thereby reducing both planktonic and biofilm-associated populations (Kim et al., 2025). Several *in vitro* and animal studies have demonstrated that phages can disrupt biofilms formed by *K. pneumoniae* and *A. baumannii*, particularly when used in combination with conventional antibiotics, resulting in enhanced bacterial clearance and reduced biofilm biomass (Ibrahim and Aranjani, 2026).

Phage-derived depolymerases have also emerged as promising anti-biofilm agents. These enzymes degrade capsular polysaccharides and EPS, facilitating improved antibiotic penetration and enhancing immune-mediated bacterial clearance (Tarasenko et al., 2025). Depolymerases have shown particular efficacy against encapsulated pathogens such as *K. pneumoniae*, where capsule degradation reduces biofilm integrity and bacterial virulence (Tarasenko et al., 2025; Jiao et al., 2025). However, despite encouraging preclinical findings, the clinical application of phage therapy and depolymerases remains limited by narrow host specificity, the potential emergence of phage-resistant bacterial variants, regulatory challenges, manufacturing standardization, and the limited availability of well-designed clinical trials (Ibrahim et al., 2025; Kim et al., 2025).

Combination therapies have emerged as a promising strategy to overcome the limitations of individual anti-biofilm interventions in MDR-VAP. Combination of antibiotics remain the foundation of treatment for severe MDR Gram-negative bacterial infections, particularly to broaden antimicrobial coverage, achieve synergistic bactericidal activity, and reduce the emergence of resistance (MacNair et al., 2024). More recently, combination approaches incorporating antibiotics with bacteriophages, nanoparticles, or matrix-degrading enzymes have demonstrated enhanced anti-biofilm activity in preclinical studies (Kim et al., 2025). Phage-antibiotic combinations can improve bacterial eradication by facilitating phage penetration into biofilms and increasing bacterial susceptibility to antibiotics, whereas nanoparticle-based delivery systems enhance antibiotic penetration and sustained release within the biofilm matrix (Wouters et al., 2024; Ramakrishnan et al., 2024). Similarly, enzymes such as DNases and glycosidases can degrade EPS, improving antibiotic access to embedded bacterial cells. Although, these combination strategies have shown encouraging synergistic effects against MDR *K. pneumoniae* and *A. baumannii*, most evidence is derived from *in vitro* and experimental animal studies. Further clinical investigations are required to establish their safety, optimal dosing strategies, and therapeutic efficacy before routine clinical implementation (Ramakrishnan et al., 2024). The emerging anti-biofilm ETTs are summarized in Table 3.

**Table 3:** Emerging anti-biofilm therapeutics for MDR-VAP treatment.

Strategy	Mechanism of action	Key quantitative findings	Advantages	Limitations	References
Silver nanoparticles (AgNPs)	ROS generation, membrane disruption, DNA damage	99–99.999% reduction in bacterial adhesion and colonization	Broad-spectrum activity, multi-target mechanism	Cytotoxicity concerns. Limited clinical validation	Lethongkam et al., 2023
Antibiotic/nanoparticle-loaded Hydrogels	Sustained antimicrobial delivery + anti-adhesion	>95% reduction in adhesion at 4 h, >99.99% at 24 h, >30-day activity	Long-term activity	Mechanical instability. Evidence mainly preclinical	Wouters et al., 2024
DNases and matrix-degrading enzymes	Degradation of eDNA and EPS components	Significant enhancement of antibiotic penetration and biofilm disruption	Targets mature biofilms	Delivery and stability challenges. Limited *in vivo* evidence	Ramakrishnan et al., 2024
Glycosidase therapy	Biofilm polysaccharide degradation	IC_50_ 2.5 μM, EC_50_ 1.9 μM	Restores antibiotic susceptibility	Pathogen-specific activity. Pre-clinical evidence only	Ramakrishnan et al., 2024
Nitric oxide-releasing systems	Biofilm dispersal and antimicrobial activity	~93% reduction in bacterial colonization within 24 h	Penetrates biofilms effectively	Release control challenges. Limited clinical validation	Aveyard et al., 2024
Antimicrobial peptides (AMPs)	Membrane disruption and matrix penetration	90–99% reduction in biofilm formation	Low resistance potential	Peptide instability. Few *in vivo* studies	Roque-Borda et al., 2021
AMP-loaded nanoparticles	Enhanced peptide delivery	~80% disruption of mature biofilms	Effective against established biofilms	Manufacturing complexity. Limited clinical evidence	Atefyekta et al., 2019
Ceragenins	Synthetic AMP mimics	Broad-spectrum anti-biofilm activity against MDR pathogens	Protease-resistant, stable	Limited clinical validation	Roque-Borda et al., 2021
Quorum sensing inhibitors	Disruption of bacterial communication	Reduced biofilm formation and virulence expression	Lower resistance pressure	Mostly *in vitro* evidence	Roy et al., 2022; Niu et al., 2024
Resveratrol	Anti-virulence and biofilm sensitization	2–4 fold reduction in eradication concentrations of disinfectants	Synergistic effects	Limited standalone efficacy. Required clinical validation	Migliaccio et al., 2024
Phage therapy	Bacterial lysis and enzymatic degradation of capsule/EPS	Significant biofilm reduction and enhanced antibiotic activity in pre-clinical studies	Pathogen specific, disrupts mature biofilms, synergistic with antibiotics	Narrow host range, phage resistance, regulatory and manufacturing challenges, limited clinical validation	Ibrahim et al., 2025; Kim et al., 2025

Emerging anti-biofilm therapies have demonstrated encouraging activity against MDR Gram-negative pathogens, particularly in reducing biofilm biomass and enhancing antibiotic penetration. Nevertheless, the majority of supporting evidence originates from *in vitro* studies, with comparatively few animal studies and limited human clinical trials. Differences in experimental biofilm models, antimicrobial concentrations, and outcome measures further affect the direct comparison between studies. While these approaches show considerable promise, their clinical utility remains uncertain until validated through well-designed clinical trials evaluating efficacy, safety, cost-effectiveness, and long-term outcomes. Although, these approaches show considerable promise, none are currently recommended in international clinical guidelines for routine management of MDR-VAP because robust clinical evidence remains limited. Their future incorporation into guideline recommendations will depend on well-designed clinical trials demonstrating safety and clinical benefit.

## 8 Future perspectives and clinical translation

Despite substantial advances in antimicrobial coatings, nanotechnology-based platforms, and biofilm-targeted therapeutics, translation of these innovations into routine clinical practice remains limited (Mishra et al., 2024). While numerous strategies have demonstrated notable efficacy in *in vitro* and preclinical models, relatively few have progressed to large-scale clinical evaluation. This translational gap reflects the complexity of biofilm-associated infections and the challenges associated with reproducing clinically relevant conditions during experimental testing (Roy et al., 2022). Biofilms formed in mechanically ventilated patients are highly heterogeneous, influenced by host factors, polymicrobial interactions, antimicrobial exposure, and the dynamic conditions of the intensive care unit environment, making successful translation from laboratory models particularly challenging (Chen et al., 2022).

One of the most promising developments is the emergence of smart and stimuli-responsive biomaterials capable of releasing antimicrobial agents in response to infection-associated triggers such as bacterial enzymes, quorum-sensing molecules, or local pH changes (Iskandar et al., 2021). Unlike conventional coatings that continuously release antimicrobial compounds, responsive systems provide targeted delivery only when bacterial colonization occurs, potentially prolonging device lifespan while minimizing selective pressure for resistance development (Rana et al., 2025). Similarly, advances in photodynamic and photocatalytic therapies offer non-antibiotic approaches capable of generating ROS that disrupt biofilms through mechanisms largely independent of conventional antimicrobial resistance pathways (Lethongkam et al., 2023). Curcumin functionalized surfaces, for example, have demonstrated approximately 95% reductions in viable biofilm-associated bacteria following light activation (Zangirolami et al., 2020).

The integration of additive manufacturing and precision medicine may further transform VAP prevention strategies. 3-D printing technologies allow the development of customized ETT with tailored surface architectures, antimicrobial incorporation, and patient-specific design characteristics (Kruse et al., 2024). Combined with advances in microbiological diagnostics and resistance profiling, such approaches may enable personalized preventive and therapeutic strategies directed against the predominant pathogens and resistance mechanisms present within individual healthcare settings (Kruse et al., 2024).

Despite these advances, several barriers continue to impede clinical implementation. Long-term biocompatibility, coating durability, manufacturing scalability, regulatory approval pathways, and cost-effectiveness remain major considerations (Chen et al., 2022). The absence of standardized biofilm models and universally accepted methods for evaluating anti-biofilm efficacy complicates direct comparison between emerging technologies and limits the generation of robust clinical evidence (Sharma et al., 2019). Future research should prioritize clinically relevant *in vivo* models, multi-center clinical trials, and combination approaches that integrate antimicrobial therapy with biofilm-targeted interventions. Such strategies are likely to be essential for overcoming the persistent challenges posed by MDR *K. pneumoniae* and *A. baumannii* biofilms in VAP (Mitache et al., 2025).

Several important research gaps must be addressed before emerging anti-biofilm therapies can be routinely incorporated into the management of MDR-VAP. First, standardized *in vitro, ex vivo*, and clinically relevant *in vivo* biofilm models are needed to enable meaningful comparison of therapeutic efficacy across studies. Second, well-designed multi-center clinical trials are required to establish the safety, efficacy, optimal dosing, and long-term outcomes of promising interventions, including bacteriophage-based therapies, nanoparticles, antimicrobial peptides, and matrix-disrupting enzymes. Third, greater emphasis should be placed on the development of combination strategies that integrate conventional antimicrobial therapy with biofilm-targeted approaches while minimizing the emergence of resistance. Lastly, advances in rapid diagnostics, biomarker-guided therapy, and precision medicine should be incorporated into future clinical studies to facilitate patient stratification and individualized treatment selection. Addressing these priorities will be essential for translating promising experimental findings into safe, effective, and clinically applicable therapies for biofilm-associated MDR-VAP.
